# Molecular Profiling of Multiplexed Gene Markers to Assess Viability of *Ex Vivo* Human Colon Explant Cultures

**DOI:** 10.1089/biores.2015.0029

**Published:** 2015-11-01

**Authors:** Janice E. Drew, Andrew J. Farquharson, Hollie Vase, Frank A. Carey, Robert J.C. Steele, Ruth A. Ross, David C. Bunton

**Affiliations:** ^1^ Metabolic Health, Rowett Institute of Nutrition and Health, University of Aberdeen, Aberdeen, Scotland.; ^2^ Ninewells Hospital and Medical School, Dundee, Scotland.; ^3^ Department of Pharmacology and Toxicology, Faculty of Medicine, University of Toronto, Toronto, Ontario, Canada.; ^4^ Biopta Ltd., Glasgow, Scotland.

**Keywords:** cell marker, gut explant, microanatomy, molecular profile, multiplex

## Abstract

Human colon tissue explant culture provides a physiologically relevant model system to study human gut biology. However, the small (20–30 mg) and complex tissue samples used present challenges for monitoring tissue stability, viability, and provision of sufficient tissue for analyses. Combining molecular profiling with explant culture has potential to overcome such limitations, permitting interrogation of complex gene regulation required to maintain gut mucosa in culture, monitor responses to culture environments and interventions. Human *ex vivo* colon explant gene expression profiles were assayed using an in-house custom-designed hCellMarkerPlex assay at culture time points 0, 1, 2, 4, and 14 h. Characteristic profiles of epithelial cell markers linked to differentiation, cellular polarization, and apoptosis were correlated with visible histochemical changes in explant epithelium during culture and tissue donors. The GenomeLab System provides effective assay of multiple targets not possible from small tissue samples with conventional gene expression technology platforms. This is advantageous to increase the utility of the *ex vivo* human colon model in applications to interrogate this complex and dynamic tissue environment for use in analytical testing.

## Introduction

Colon cell monocultures used extensively as an *in vitro* model system of the human gut provide limited information, failing to provide physiologically relevant data, or insights into the complex interactions between different cell types that comprise the colon mucosa.^1^ Colon cell lines are typically transformed, lack characteristic cellular architecture, exhibit different gene expression profiles in comparison with normal and tumor cells in the human body.^1,2^ *Ex vivo* cultured human colon tissue presents an alternative model system that is physiologically relevant to study human biology to generate data on metabolic responses and signaling pathways.^3,4^

Cultured explants consist of mucosa, a single layer of epithelial cells, the lamina propria and the muscularis mucosae.^5,6^ The epithelium consists of columnar epithelial cells (colonocytes), mucus-producing goblet cells, and scattered enteroendocrine cells, which form thin tubular glands known as crypts.^5,6^ The epithelial layer is perpetually renewed as a consequence of regulated proliferation of stem cells at the base of each crypt.^7^ The epithelium overlies the lamina propria, a cell-rich connective tissue containing fibroblasts, macrophages, lymphocytes, eosinophilic leukocytes, mast cells, and blood vessels.^6^ The epithelium and lamina propria are surrounded by a continuous sheet of smooth muscle, the muscularis mucosae.^6^ These small tissue explants (typically 20–30 mg) limit comprehensive molecular analysis by conventional technology platforms. Monitoring donor variation and establishing normal as distinct from dysplastic tissue is important since tissue specimens are often obtained from patients attending for colectomy as a treatment for benign polyps or colorectal adenocarcinoma. This report investigates the application of in-house custom-designed gene expression assays^8,9^ to establish normal molecular profiles of human colon tissue explants, identify donor characteristics, and monitor cellular processes and aspects of tissue stability and viability within colon explants during culture.

## Materials and Methods

### Human colon tissue

Colectomy tissue was obtained through the Tayside Tissue Bank (Dundee, Scotland) from patients attending for colectomy as a treatment for benign polyps or colorectal adenocarcinoma (Ninewells Hospital, Dundee, Scotland). All patients consented for research use of tissues using the forms approved by the Tayside Local Research Ethics Committee through the Tayside Tissue Bank. Following visual assessment by a qualified pathologist, a piece of normal colon tissue comprising all tissue layers (mucosa, submucosa, muscle, subserosa, and serosa) was removed and placed in physiological saline solution (PSS; 119 mM NaCl, 4.7 mM KCl, 1.2 mM MgSO_4_, 24.9 mM NaHCO_3_, 1.2 mM KH_2_PO_4_, 2.5 mM CaCl_2_, 11.1 mM glucose, pH = 7.6, 4°C) before preparation explants.

### Explant preparation and culture

Mucosa was dissected in PSS at 4°C and explants (*n* = 14) prepared (3 × 3 mm in duplicate) using a scalpel. Explants were placed, mucosal layer uppermost, on wire mesh grids in six-well culture plates (Nunc GmbH & Co. KG) with ∼3.5 mL of culture media, RPMI (Sigma-Aldrich), and 1% fetal bovine serum [Cambrex (UK &Eire) Corp] to a level barely covering the explant. Culture plates were placed in a modular incubator chamber (MIC) (Billups-Rothenberg, Inc.) that was sealed and gassed with 95% O_2_/5% CO_2_ for 10 min. Culture dishes in the MIC were continuously rocked (Grant BFR25 rocker; Cambridge Ltd) inside a Forma Scientific CO_2_ incubator (Forma Scientific UK Ltd) at 37°C. At culture time points 0, 1, 2, 4, and 14 h, duplicate explants were frozen in dry ice and stored at −80°C until use. The MIC was flushed with 95% O_2_/5% CO_2_ for 10 min at each culture time point.

### Histological analysis

Cryostat tissue sections (10 μm) were Hematoxylin and eosin (H&E) stained and viewed using a Leica DMR microscope [Leica Microsystem (UK) Ltd] and imaged using a QImaging QICAM Fast 1394 Digital CCD Camera (QImaging) and QCapture Pro 6.0 Software (QImaging).

### hCellMarkerPlex profiling of human colon explants

Colon explant total RNA (50 ng in triplicate) samples (0, 1, 2, 4, and 14 h culture) were extracted and assayed using the hCellMarkerPlex and the GenomeLab GeXP Start Kit (Beckman Coulter) as described previously.^8,9^ Yields were typically ∼20 μg with RIN values from 8 to 10.

### Statistical analysis

Principal component analysis (PCA) was performed using SIMCA-P +12.0 software (MKS Instruments UK Ltd) on normalized hCellMarkerPlex assay data from colon explants (*n* = 14 tissue donors) to assess expression patterns at culture time points tested and differences between tissue donor samples. Analysis of variance (ANOVA) was performed on normalized gene expression data, blocked for tissue donor with time points as treatment, using GenStat^®^ 13th Edition (VSN International, Ltd.). A *post hoc* Bonferroni correction for multiple comparisons of time points within an ANOVA was applied (significance level 0.05). ANOVA was conducted on a log scale if data were skewed.

## Results and Discussion

**FIG. 1.**
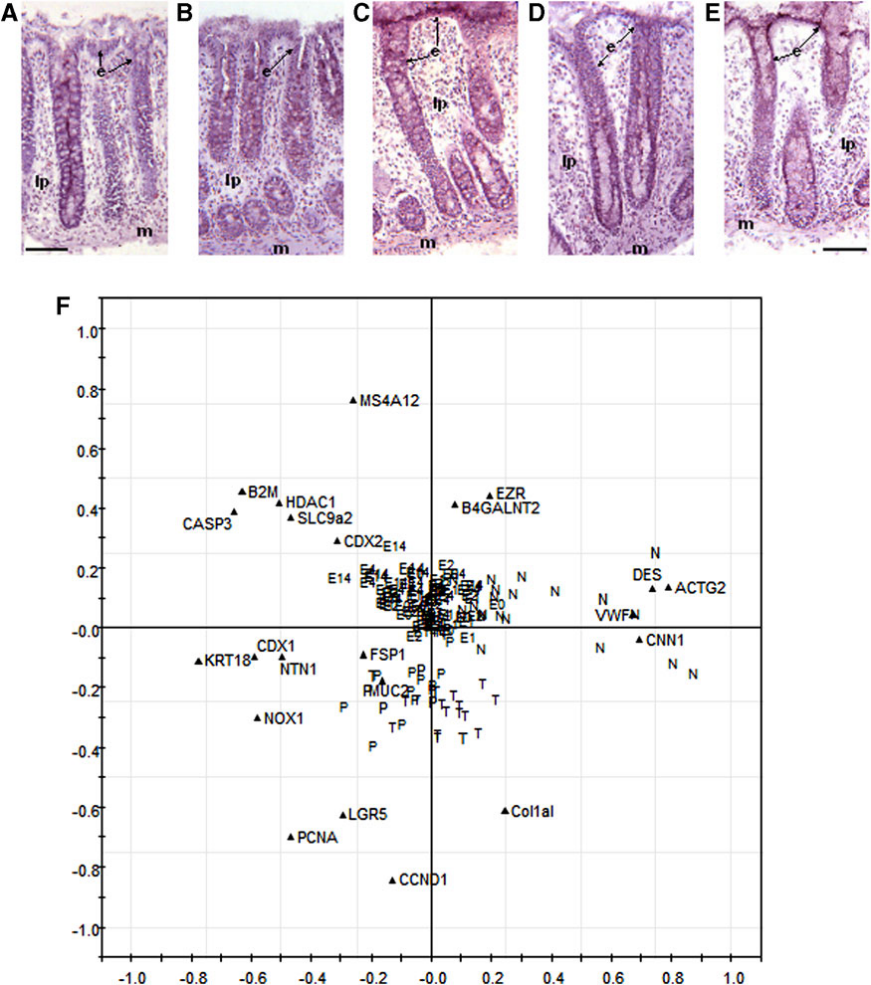
Histological features of normal colon tissue in explant culture at 0 h **(A)**, 1 h **(B)**, 2 h **(C)**, 4 h **(D)**, and 14 h **(E)**. Frozen tissue is Hematoxylin and eosin stained. Scale bar = 100 μm. **(F)** Biplot of the first two principle components (PCA plot) of hCellMarkerPlex gene expression data. The hCellMarkerPlex was applied to total RNA from colon explants cultured at 0 h (E0), 1 h (E1), 2 h (E2), 4 h (E4), and 14 h (E14) and compared with hCellMarkerPlex data from a previous study of human colon biopsy tissues, normal (N), adenomatous polyp (P), and carcinoma (T).^13^ Data have been normalized to *UBE2D2*. The PCA plot reveals clustering of colon explants and normal tissue indicating a greater similarity in gene expression profiles compared with the greater divergence in gene expression in adenomatous polyp and carcinoma. Gene targets are identified by name and location designated by (▲). The position of the gene targets signifies levels of expression characterizing the tissue types. e, epithelium; lp, lamina propria; m, muscularis mucosa; PCA, principal component analysis.

Microanatomical analysis revealed normal histological epithelium and crypt structure that was maintained between 0 and 4 h culture time points (Fig. 1A–D). At 14 h, lamina propria, muscularis mucosae, and surface epithelium were still clearly visible, but loss of cell density in the lamina propria and reduced epithelial cell volume were observed (Fig. 1E). This was supported by the PCA biplot of normalized hCellMarkerPlex gene expression data (Fig. 1F). Colon explant profiles exhibited a gene expression pattern characteristic of normal tissue when compared to data from a previous study of normal colon, adenomatous polyp, and carcinoma tissues^8^ (Fig. 1F). Higher expression levels of epithelial markers *MS4A12*, *EZR*, and differentiation marker *B4GANLT2*, and lower expression levels of stem cell marker *LGR5*, proliferation markers *PCNA* and *CCND1*, and fibroblast marker *COL1A1* (Fig. 1F) are associated with cultured explants and characterize normal tissue as opposed to colon adenomatous polyp or carcinoma tissues (Fig. 1F).

Microanatomical changes at 14 h were associated with increased expression of *EZR*, *HDAC1*, *KRT18*, *B2M* (a component of the major histocompatibility complex class I molecules),^10^ and *CASP3* (an apoptotic marker).^11,12^ Elevated *EZR* may be a response to restore loss of epithelial stability as *EZR* is required to maintain a stable normal colon epithelium.^13^ Increased *HDAC1* implies changes in gene regulation^14^ within the explant during culture. Elevated *CASP3* may indicate the induction of apoptosis.^12,15^ Four of the gene targets displaying the highest degree of expression changes with time in culture, *KRT18*,^16^ *SLC9A2*,^17^ *EZR,*^13^ and *ACTG2*^13^ are implicated in key roles in epithelial cell polarization. Little is known about epithelial cell polarization and complex cell–cell interactions within the human colon since it is difficult to recapitulate the dynamic transcriptional program of complex tissues using cell monocultures *in vitro*.^1^ Custom multiplexes can be designed to facilitate assay of small tissue specimens that specifically target components of signaling pathways to more fully capture this information.

**FIG. 2.**
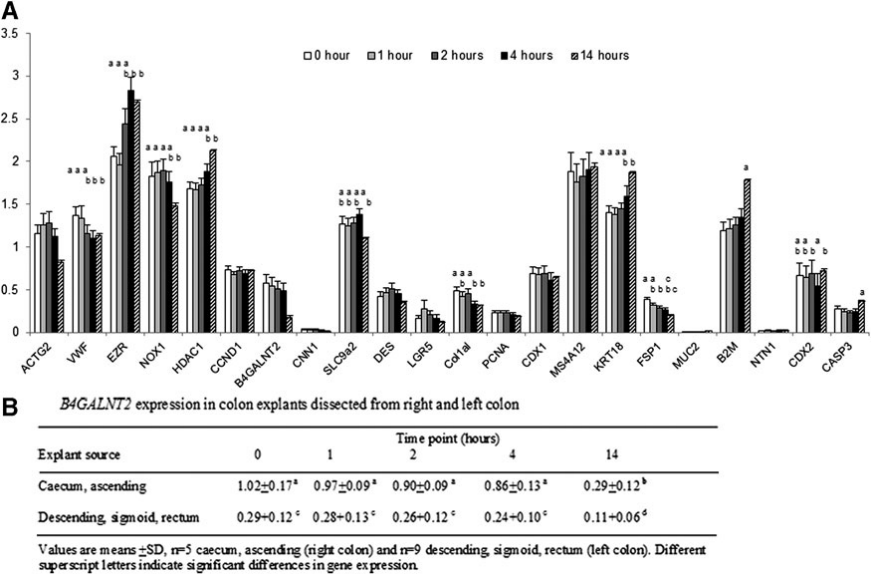
**(A)** Relative gene expression levels in human colon explants (*n* = 14) generated using the hCellMarkerPlex assay. The hCellMarkerPlex assay was applied to assess gene expression profiles of colon explant total RNA (50 ng in triplicate) samples extracted from cultured colon explants at 0, 1, 2, 4, and 14 h. The percentage of CV for each gene was calculated. The percentage of CV of 10% or less was achieved consistently for 20 of the genes within the hCellMarkerPlex (*ACTG2*, *VWF*, *EZR*, *NOX1*, *HDAC1*, *UBE2D2*, *CCND1*, *B4GALNT2*, *SLC9A2*, *DES*, *LGR5*, *COL1A1*, *PCNA*, *CDX1*, *MS4A12*, *KRT18*, *FSP1*, *B2M*, *CDX2*, and *CASP3*) as well as for the internal reference marker *Kan(r)*. Low expressers (*CNN1*, *MUC2*, and *NTN1*) exhibited more variable percentage of CV ranging from 10% to 25%. GeNorm (http://medgen.ugent.be/genorm/) identified *UBE2D2* as a consistently stable reference gene and data were thus normalized to *UBE2D2.* ANOVA blocked for tissue donor, with time points as treatment factors, was applied. A *post hoc* Bonferroni correction was applied and significant differences in the expression of each gene target between the culture time points tested is indicated by unique letters above each bar (*p* < 0.05). **(B)** *B4GALNT2* expression in colon explants dissected from right and left colon. CV, coefficient of variation.

*NOX1*, *B4GALNT2*, *SLC9A2*, *COL1A1*, *FSP1*, and *VWF* were reduced at 14 h (Fig. 2A). *NOX1* and *SLC9A2* regulates cellular pH and sodium transport across the apical membrane.^17–19^ Decreased *B4GALNT2* (Fig. 2), a colon epithelial goblet cell differentiation marker,^20^ coincides with loss of epithelial cell volume. *B4GALNT2* expression levels correspond with reports of a proximal–distal gradient of expression,^20,21^ with *B4GALNT2* expression lower in the sigmoid and rectum compared to the cecum and ascending colon (Fig 2B). However, it was apparent that decreased *B4GALNT2* expression was observed in all 14 h explants regardless of tissue location origin (Fig. 2B). *COL1A1* and *FSP1*, markers of fibroblasts within the lamina propria, produce collagen.^22^ Decreased *COL1A1* and *FSP1* were apparent throughout the culture (Fig. 2A), potentially a consequence of visible cell loss within the lamina propria (Fig. 1E). Donor variation in *MUC2*, *NTN1*, and *CNN1* was largely attributed to low expression levels. Variation in *CNN1* a smooth muscle marker^23,24^ is likely a consequence of slight variation in smooth muscle attached to explants (Fig. 1A–E).

Eight gene markers associated with proliferation (*PCNA*, *CCND1*, *MS4A12*), differentiation (*CDX1*), apoptosis (*NTN1*), structural (*CNN1*, *DES*), and the stem cell marker *LGR5*, were not significantly altered during explant culture (Fig. 2A). *MS4A12* is a CDX-regulated colon-specific epithelial marker that is specifically located in luminal surface epithelium^25^ and is linked to regulation of proliferation.^26^ *CDX1*, a member of the homeobox genes of the *caudal* family, is involved in epithelial differentiation.^27^ *DES* is a muscle cell marker.^23^ *LGR5* is a colon stem cell marker, expressed within 4–6 cells at the base of each crypt,^28^ demonstrating the sensitivity of the hCellMarkerPlex. The lack of significant changes in the expression of these gene markers implies that there is no increased cell proliferation and differentiation on introduction of the explants to the culture system.

GeXP technology permits effective gene expression profiling in small tissue samples that is not possible using conventional technology platforms. The molecular profiles permit monitoring of tissue stability, viability, and donor characteristics. This is advantageous to increase utility of the *ex vivo* human colon model to interrogate this complex and dynamic tissue environment to generate physiologically relevant data on gene networks or signaling pathways and their roles within different cells and tissues. Development of further custom-designed assays will be a valuable tool for investigation of gene regulation in the very small tissue samples used for *ex vivo* colon explant culture to identify tissue reponses to interventions.

## Abbreviations Used

ANOVA: analysis of variance
MIC: modular incubator chamber
PCA: Principal component analysis
PSS: physiological saline solution

## References

[1] Ma C Animal models of disease. Mod Drug Discov. 2004;7:30–36

[2] Talmadge JE, Singh RK, Fidler IJ, et al. Murine models to evaluate novel and conventional therapeutic strategies for cancer. Am J Pathol. 2007;170:793–804 17322365 10.2353/ajpath.2007.060929 PMC1864878

[3] Pritchett CJ, Senior PV, Sunter JP Cell proliferation in human colorectal mucosa in organ culture: the early adaptive changes. J Anat. 1985;141:171–179 4077714 PMC1166398

[4] Resau JH, Sakamoto K, Cottrell JR, et al. Explant organ culture: a review. Cytotechnology. 1991;7:137–149 1368116 10.1007/BF00365924

[5] Rao JN, Wang JY Regulation of Gastrointestinal Mucosal Growth. Morgan & Claypool Life Sciences: San Rafael, CA; 2010 21634069

[6] Keshav S The Gastrointestinal System at a Glance. Wiley-Blackwell: United Kingdom; 2003

[7] Sato T, Vries RG, Snippert HJ, et al. Single Lgr5 stem cells build crypt-villus structures in vitro without a mesenchymal niche. Nature. 2009;459:262–265 19329995 10.1038/nature07935

[8] Drew JE, Mayer C, Farquharson AJ, et al. Custom design of a GeXP multiplexed assay used to assess expression profiles of inflammatory gene targets in normal colon, polyp, and tumor tissue. J Mol Diagn. 2011;13:233–242 21354059 10.1016/j.jmoldx.2010.10.001 PMC3128578

[9] Drew JE, Farquharson AJ, Mayer CD, et al. Predictive gene signatures: molecular markers distinguishing colon adenomatous polyp and carcinoma. PLoS One. 2014;9:e113071 25423035 10.1371/journal.pone.0113071 PMC4244109

[10] Iwata K, Matsuura T, Sakurai K, et al. High-resolution crystal structure of β2-microglobulin formed at pH 7.0. J Biochem. 2007;142:413–419 17646174 10.1093/jb/mvm148

[11] Bortner CD, Cidlowski JA Apoptotic volume decrease and the incredible shrinking cell. Cell Death Differ. 2002;9:1307–1310 12478467 10.1038/sj.cdd.4401126

[12] Porter AG, Jänicke RU Emerging roles of caspase-3 in apoptosis. Cell Death Differ. 1999;6:99–104 10200555 10.1038/sj.cdd.4400476

[13] Cant SH, Pitcher JA G protein-coupled receptor kinase 2-mediated phosphorylation of Ezrin is required for G protein-coupled receptor-dependent reorganization of the actin cytoskeleton. Mol Biol Cell. 2005;16:3088–3099 15843435 10.1091/mbc.E04-10-0877 PMC1165394

[14] Pflum MKH, Tong JK, Lane WS, et al. Histone deacetylase 1 phosphorylation promotes enzymatic activity and complex formation. J Biol Chem. 2001;276:47733–47741 11602581 10.1074/jbc.M105590200

[15] Riedl SJ, Shi Y Molecular mechanisms of caspase regulation during apoptosis. Nature Rev Mol Cell Biol. 2004;5:897–907 15520809 10.1038/nrm1496

[16] Owens DW, Lane EB Keratin mutations and intestinal pathology. J Pathol. 2004;204:377–385 15495267 10.1002/path.1646

[17] Ghishan FK, Knobel SM, Summar M Molecular cloning, sequencing, chromosomal localization, and tissue distribution of the human Na^+^/H^+^ exchanger (SLC9A2). Genomics. 1995;30:25–30 8595899 10.1006/geno.1995.0004

[18] Henderson LM, Chappell JB, Jones OTG Internal pH changes associated with the activity of NADPH oxidase of human neutrophils. Further evidence for the presence of an H^+^ conducting channel. Biochem J. 1998;251:563–567 2456757 10.1042/bj2510563 PMC1149038

[19] Malakooti J, Dahdal RY, Dudeja PK, et al. The human Na^+^/H^+^ exchanger NHE2 gene: genomic organization and promoter characterization. Am J Physiol Gastrointest Liver Physiol. 2001;280:G763–G773 11254504 10.1152/ajpgi.2001.280.4.G763

[20] Malagolini N, Dall'Olio F, Serafini-Cessi F UDP-GalNAc:NeuAcα2,3Galβ-R (GalNAc to Gal) β1,4-N-acetylgalactosaminyltransferase responsible for the Sda specificity in human colon carcinoma CaCo-2 cell line. Biochem Biophys Res Commun. 1991;180:681–686 1953740 10.1016/s0006-291x(05)81119-2

[21] Morton JA, Pickles MM, Vanhegan RI The Sda antigen in the human kidney and colon. Immunol Invest. 1988;17:217–224 3044991 10.3109/08820138809052961

[22] Rossert J, Terraz C, Dupont S Regulation of type I collagen genes expression. Nephrol Dialysis Transplant. 2000;15 S6:66–68 10.1093/ndt/15.suppl_6.66 11143996

[23] Paulin D, Li Z Desmin: a major intermediate filament protein essential for the structural integrity and function of muscle. Exp Cell Res. 2004;301:1–7 15501438 10.1016/j.yexcr.2004.08.004

[24] Takahashi K, Tazunoki T, Okada T, et al. The 5′-flanking region of the human smooth muscle cell calponin gene contains a cis-acting domain for interaction with a methylated DNA-binding transcription repressor. J Biochem. 1996;120:18–21 8864837 10.1093/oxfordjournals.jbchem.a021382

[25] Koslowski M, Tureci O, Huber C, et al. Selective activation of tumor growth-promoting Ca^2^ channel MS4A12 in colon cancer by caudal type homeobox transcription factor CDX2. Mol Cancer. 2009;8:77 19781065 10.1186/1476-4598-8-77 PMC2759907

[26] Koslowski M, Sahin U, Dhaene K, et al. MS4A12 is a colon-selective store-operated calcium channel promoting malignant cell processes. Cancer Res. 2008;68:3458–3466 18451174 10.1158/0008-5472.CAN-07-5768

[27] Bonner CA, Loftus SK, Wasmuth JJ Isolation, characterization, and precise physical localization of human cdx1, a caudal-type homeobox gene. Genomics. 1995;28:206–211 8530027 10.1006/geno.1995.1132

[28] Barker N, Van Es JH, Kuipers J, et al. Identification of stem cells in small intestine and colon by marker gene Lgr5. Nature. 2007;449:1003–1007 17934449 10.1038/nature06196

